# The Current Landscape of Prostate-Specific Membrane Antigen (PSMA) Imaging Biomarkers for Aggressive Prostate Cancer

**DOI:** 10.3390/cancers16050939

**Published:** 2024-02-26

**Authors:** Haidar Al Saffar, David C. Chen, Carlos Delgado, Jacob Ingvar, Michael S. Hofman, Nathan Lawrentschuk, Marlon Perera, Declan G. Murphy, Renu Eapen

**Affiliations:** 1 Division of Cancer Surgery, Peter MacCallum Cancer Centre, Melbourne, VIC 3052, Australia; david.chen@petermac.org (D.C.C.); jacob.ingvar@petermac.org (J.I.); lawrentschuk@gmail.com (N.L.); marlonlperera@gmail.com (M.P.); declan.murphy@petermac.org (D.G.M.); renu.eapen@petermac.org (R.E.); 2 Prostate Cancer Theranostics and Imaging Centre of Excellence, Molecular Imaging and Therapeutic Nuclear Medicine, Cancer Imaging, Peter MacCallum Cancer Centre, Melbourne, VIC 3052, Australia; michael.hofman@petermac.org; 3 Department of Surgery, Austin Health, Heidelberg, VIC 3084, Australia; 4 School of Medicine and Health Sciences, Tecnologico de Monterrey, Monterrey 64849, Mexico; carlos_delrdz@hotmail.com; 5 Sir Peter MacCallum Department of Oncology, University of Melbourne, Melbourne, VIC 3052, Australia; 6 Department of Surgery (Urology), Royal Melbourne Hospital, Melbourne, VIC 3052, Australia; 7 EJ Whitten Prostate Cancer Research Centre, Epworth Hospital, Richmond, VIC 3121, Australia

**Keywords:** prostate-specific membrane antigen, PSMA PET/CT, aggressive prostate cancer, uro-oncology, biochemical recurrence

## Abstract

**Simple Summary:**

The review explores the critical role of prostate-specific membrane antigen (PSMA) PET/CT imaging in diagnosing, staging, and treating prostate cancer. PSMA PET/CT offers superior diagnostic capabilities for identifying prostate cancer’s spread, with potential as a prognostic indicator for the disease’s recurrence and survival. It highlights PSMA’s variability in expression, impacting personalised treatment plans, notably in radioligand therapy with [^177^Lu] Lu-PSMA-617. This technology enhances treatment strategies, improves outcomes, and reduces unnecessary interventions, marking a significant advancement in personalised prostate cancer management.

**Abstract:**

The review examines the vital role of prostate-specific membrane antigen (PSMA) positron emission tomography/computed tomography (PET/CT) in the diagnosis, staging, and treatment of prostate cancer (PCa). It focuses on the superior diagnostic abilities of PSMA PET/CT for identifying both nodal and distant PCa, and its potential as a prognostic indicator for biochemical recurrence and overall survival. Additionally, we focused on the variability of PSMA’s expression and its impact on personalised treatment, particularly the use of [^177^Lu] Lu-PSMA-617 radioligand therapy. This review emphasises the essential role of PSMA PET/CT in enhancing treatment approaches, improving patient outcomes, and reducing unnecessary interventions, positioning it as a key element in personalised PCa management.

## 1. Introduction

Aggressive prostate cancer (PCa) remains a challenge, with high rates of recurrence, morbidity, and mortality despite early diagnosis [1]. The emerging use of prostate-specific membrane antigen (PSMA) positron emission tomography/computed tomography (PET/CT) in PCa imaging provides unprecedented visualisation of cancer on a molecular level [2].

PSMA is an ideal target in PCa, as PSMA’s expression increases with aggressive diseases [3]. However, PSMA is not PCa-specific, and its physiological expression can be seen in the renal cortex, urine, salivary and lacrimal glands, while mild to moderate activity is seen in the liver, spleen, and gastrointestinal tract [4,5]. Further, pathologic expression in tumours include renal cell, gastric, colorectal, breast, and thyroid cancers [5], with a substantial correlation between PSMA and vascular endothelial growth factor having been observed, indicating the potential of PSMA as a marker of angiogenesis [6]. It should be noted that in neuroendocrine differentiation, PSMA’s expression is significantly repressed and will require other forms of imaging for accurate cancer staging [7].

**Figure 1:**
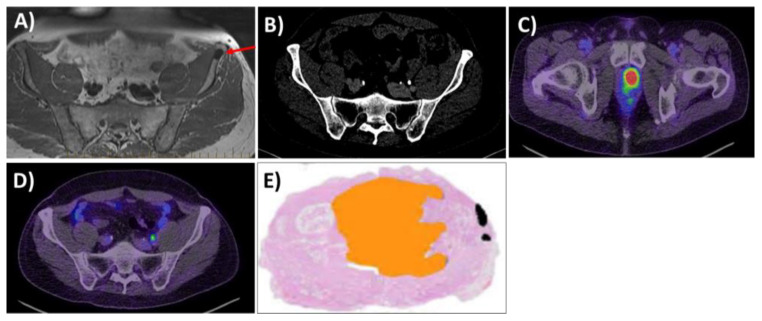
Clinical case from routine practice at the Peter MacCallum Cancer Centre (PMCC), where the use of PSMA PET/CT had dramatically changed therapeutic interventions. The patient presented with a PSA of 45 and a left Prostate Imaging Reporting and Data System (PIRADS) 5 lesion on MRI, with the biopsy confirming left GGG2 disease. The MRI and CT scans also showed a suspicious sclerotic lesion in the left iliac crest, which was concerning for bony metastases (**A**,**B**). The red arrow highlights the bony lesion in the left iliac crest. The bone appeared to show no apparent disease. Conversely, the PSMA PET/CT showed high PSMA avidity, with a maximum SUV (SUVMax) of 21.9 in the left prostate lesion and no avidity within the previously identified bone lesion (**C**,**D**). The patient proceeded to have a radical prostatectomy (RP) for localised high-risk PCa (**E**). At 3 years after the curative treatment, the patient’s PSA remained undetectable.

In contemporary practice, PSMA has several clinical implications, most pertinently in the setting of diagnostics and in the use of PSMA-based theranostic agents. In the diagnostic setting, the ubiquitous PSMA tracers are [^68^Ga]Ga-PSMA-11 and [^18^F]DCFPyL. While no high-quality head-to-head comparisons have been conducted between the PSMA tracers, they are considered to be generally comparable in clinical settings, apart from minor metabolic differences [8,9]. Conventional imaging (CI) of PCa comprises CT, bone scans, and magnetic resonance imaging MRI [10]. Randomised trials have demonstrated the superiority of PSMA PET/CT over CI for the diagnosis of nodal and distant disease [2,11,12]. Hofman et al. demonstrated a 27% greater accuracy for PSMA PET/CT over CI in the proPSMA study. Moreover, the advantages of PSMA PET/CT include fewer equivocal results and high reporter agreement [2]. For nodal staging, PSMA PET/CT has a sensitivity and specificity of 75% and 99% on a per LN basis, and 77% and 97% on a per patient basis, respectively [13]. PSMA PET/CT has a significant impact on therapeutic management options following a diagnosis of PCa [14] (Figure 1).

**Figure 2:**
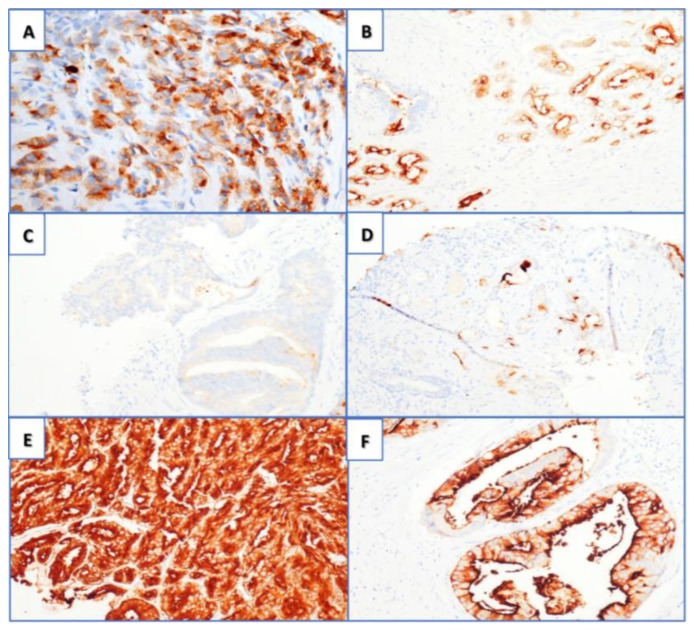
PSMA immunohistochemistry (magnification 20×). (**A**) Cytoplasmatic immunoreaction; (**B**) membranous positivity. Visual scores for PSMA positivity: (**C**) score 0, (**D**) score 1+, (**E**) score 2+, and (**F**) score 3+ (both cytoplasmic and membranous positivity) [20]. Reused under open access Creative Commons CC BY 4.0 license.

PSMA’s expression increases from locally advanced metastatic hormone-sensitive prostate cancer (mHSPC) to metastatic castration-resistant prostate cancer (mCRPC) [15]. High PSMA expression is also associated with a greater risk of relapse following a prostate biopsy or radical prostatectomy (RP), independent of the prostate-specific antigen (PSA) or Gleason grade group (GGG) [13]. Comparisons between PSMA PET/CT and MRI found that [^68^Ga] Ga-PSMA-11 had a higher sensitivity and comparable specificity for staging preoperative LN metastases in intermediate and high-risk PCa [16,17]. Despite the notable advantages, small metastatic deposits under the spatial resolution of PET (~3–5 mm) may still be missed, suggesting that PSMA PET/CT cannot replace diagnostic extended pelvic LN dissection (ePLND) at this stage [18]. Another common pitfall is the detection of small LNs in the para-aortic region with activity often representing the coeliac ganglia. These run along the aorta and do not represent metastatic disease often, with a standard uptake value (SUV) of <4 [19]. PSMA’s expression can also be visually scored through immunohistochemistry. Figure 2 depicts the grading of PSMA’s expression under microscopic intensification [20].

In this review, we discuss the current state of PSMA’s expression in PCa by assessing the clinical applications and impact on diagnosis, staging, and treatment. The use of imaging biomarkers and PSMA’s expression holds significant promise in improving the management and outcomes of patients with PCa.

## 2. Correlation between PSMA’s Expression and Prognosis in Localised Disease

### 2.1. Low or Negative Expression of PSMA

Approximately 3.4% of patients with intermediate to high-risk PCa who undergo PSMA PET/CT prior to definitive treatment have a negative prostate expression of PSMA [21].

PSMA PET/CT positivity of the primary prostate tumour can be defined by the maximum standard uptake value (SUVMax), which measures the absorbed radiation. SUVMax is highly dependent on increased immunohistochemical (IHC) expression of PSMA protein (Figure 2) [20,21]. Rüschoff described an IHC PSMA-negative tumour area of ≥20% in the RP specimen as having the strongest association with negative PSMA PET/CT [22]. Applying this ≥20% cut-off resulted in 89% sensitivity and 86% specificity for a negative PSMA PET/CT scan. A cut-off of ≥90% PSMA-positive cells for PET positivity was proposed in another study [23]. An infiltrative growth pattern, smaller tumour size, and lower tumour grades are also associated with lower PSMA uptake of primary PCa [22].

Contrary to previous theories suggesting that negative PSMA-expressing tumours are associated with neuroendocrine differentiation and worse prognosis, Veerman et al. results demonstrated these patients have similar clinical and pathological characteristics to patients with PSMA-expressing PCa, suggesting, in turn, that treatment for curative intent should not be withheld on these grounds [21,24]. Biochemical recurrence (BCR)-free survival (BCR-FS) among these patients is similar to that in patients with PSMA-expressing PCa [25]. More data are needed to better understand the long-term clinical and prognostic implications of these PCa tumours with negative PSMA uptake.

More recently, the role of PSMA PET/CT to improve risk stratification in active surveillance (AS) patients has been evaluated. In a single-centre prospective cohort study (PASPoRT), Heetman et al. performed an additional [^68^Ga] Ga-PSMA-11 PET/CT and targeted biopsies of all PSMA lesions with a SUVMax of ≥4 that were not covered by previous biopsies [26]. All participants had previously been enrolled in an AS program and had undergone a prebiopsy MRI and a targeted biopsy for visualised lesions. After the PSMA-targeted biopsies, 9% of the patients were upgraded. A systematic review exploring the use of PSMA PET/CT in low- to intermediate risk PCa suggested it can improve risk stratification by detecting MRI-occult lesions and identifying patients at risk of pathological upstaging [27]. Further prospective studies are needed to prove its efficacy in this group of patients.

### 2.2. Heterogeneity in PSMA’s Expression

Heterogeneity in PSMA exists on an intralesional and interlesional level, as seen by variations in PSMA’s expression in local tumours and metastatic lesions [28]. Comprehending this aspect is essential to enhancing patient selection for PSMA-targeted therapies. The efficacy of [^177^Lu]Lu-PSMA-617 therapy is more frequently observed when patient selection is guided by PSMA PET/CT imaging.

The use of contemporaneous PSMA PET/CT and FDG PET/CT has been described by Hofman et al. to identify patients who have a high expression of PSMA at all disease sites and are most likely to benefit from [^177^Lu]Lu-PSMA-617 treatment [29]. Substantial evidence has indicated that patients exhibiting low expression or discordant FDG-avid disease and who are undergoing PSMA-targeted therapy are likely to have an unfavourable prognosis [30].

PSMA’s expression evolves with the disease’s progression. Paschalis et al. reported that patients with mCRPC and DNA-repair-defective tumours had higher expression of PSMA [31]. They hypothesised that deleterious DNA damage repair aberrations are associated with replication stress, which increase cellular demand for folate and glutamate to further stimulate DNA synthesis and repair. Future clinical trials will examine whether PSMA-targeted treatments can improve the response for tumours with defective DNA damage repair.

Prior research has indicated that the inhibition of androgen receptors may lead to an increase in PSMA’s expression in PCa cells [32,33]. More recently, an international multicentre retrospective study reported that the commencement of androgen receptor pathway inhibitors (ARPI) in mCRPC patients is associated with only a slight increase in the whole-body expression of PSMA but without a strong effect on the whole-body PSMA SUVMax or mean SUV [34]. Numerous mechanisms of PSMA’s expression remain the subjects of ongoing research. Comprehending these mechanisms is crucial for identifying patients who are most likely to benefit from PSMA-targeted therapies.

### 2.3. Expression of PSMA and Histological Correlations: Pretreatment Biomarker

Increasing research interest is now focused on establishing the benefit of PSMA PET/CT prior to definitive treatment in PCa, with the aim of creating a more nuanced risk-stratification system [2,35,36]. While no consensus on the SUVMax cut-off has been established to predict malignancy or clinically significant prostate cancer (csPCa), studies have reported a notable correlation between the percentage of IHC expression of PSMA in the PCa cell membranes and a higher malignancy grade [22].

Building on this knowledge, Uprimny et al. identified higher Gleason scores in prostate biopsies with increased SUVMax [37]. Subsequently, Xue et al. studied the association between prostate SUVMax in intermediate risk PCa patients and the final RP pathology [38]. From a cohort of 220 patients, SUVMax was higher in all GG4 subgroups and was an independent predictor of the GG4 pattern according to the RP histopathology. A SUVMax cut-off of 5.4 yielded a sensitivity and specificity of 57% and 89% for predicting >50% of GG4 per segment at the final pathology. An SUVMax cut-off of 4.5 had a sensitivity and specificity of 58 and 85% for predicting >20% GGG4 per segment. Moreover, DeMirci et al. compared preoperative SUVMax with histopathology in RP specimens [39]. The study identified a cut-off value of 9.1 to predict GGG3 or higher after RP, with a rate of 62.5%. Roberts and colleagues confirmed these results in a similar study, suggesting that [^68^Ga] Ga-PSMA-11 PET/CT SUVMax predicts adverse pathological outcomes in RP specimens, and in upgrading GGG1 and −2 to GGG3 [40]. Other high-risk PCa features such as a cribriform pattern in the RP specimen have also been correlated with high SUVMax at preoperative staging [20].

More recently, the PRIMARY trial, a prospective multicentre Phase II imaging trial, evaluated the potential of PSMA PET/CT for the diagnosis of intraprostatic malignancy in men with an MRI Prostate Imaging–Reporting and Data System (PIRADS) score of 2–5 [11]. The trial identified the similar sensitivity and specificity of PSMA PET/CT and MRI for csPCa. The combination of both demonstrated a synergistic effect, showing improved sensitivity and higher negative predictive value than MRI alone [11]. There was a strong association between the intensity of PSMA and higher GG. All men with an SUVMax of ≥12 had csPCa on biopsy, independent of the MRI findings [11]. A specificity of 100% was found in men with PIRADS 4–5 and an SUVMax of ≥9. An important advantage of PSMA PET/CT in this study was in men with negative or equivocal MRI. On biopsy, 28% of men with PIRADS 2 and 47% with PIRADS 3 had csPCa, with 90% of these malignancies identified by PSMA PET/CT. Additionally, among patients with PIRADS 2–3 and a negative PSMA PET/CT, 91% did not present with csPCa upon biopsy [11].

An ongoing multicentre, two-arm, randomised controlled Phase III trial currently recruiting patients, the PRIMARY 2 trial (NCT05154162), will test the additive value of PSMA PET/CT for PCa diagnoses in men with negative or equivocal MRI [41]. The authors hypothesise that the trial findings may provide the advantage of reducing unnecessary biopsies in patients with PIRADS 2–3 [41].

These studies support the utility of PSMA PET/CT for risk stratification of PCa and subsequent decision making. It is important to be aware that specific intensity values are not directly generalizable to PET scans based on other PSMA radiotracers, and the calculated SUVMax may change depending on the PET scanners’ manufacturers and calibration. The recently developed PRIMARY score incorporates the intraprostatic pattern and the intensity of [^68^Ga] Ga-PSMA-11 PET/CT to diagnose csPCa with higher accuracy and reproducibility across a range of PET cameras and PSMA ligands.

### 2.4. Correlation between Pretreatment PSMA PET and BCR

Quantitative PET imaging parameters have been explored as potential prognostic biomarkers for BCR and overall survival (OS); however, studies have reported equivocal findings. In the largest retrospective cohort of 848 patients with biopsy-confirmed PCa receiving preoperative staging PSMA PET/CT prior to RP by Roberts et al., SUVMax was identified as a predictor of BCR, independent of the International Society of Urological Pathologists (ISUP) grade group (GG) [40]. With a median follow-up of 41 months, BCR-FS was reduced in patients with higher SUVMax, according to both continuous and quartile-based measures [40]. In addition, the investigators identified similar trends once the quartiles were stratified on the basis of the ISUP GG, suggesting that SUVMax is an independent predictor of the disease’s recurrence [39].

PSMA’s expression has been identified to be an independent risk stratifier of outcomes at the initial diagnosis [42]. Comparatively, in a cohort of 77 biopsy-confirmed patients conducted by Qiu et al. with a medium follow-up of 25 months, BCR was better predicted by a combination of SUVMax, the maximum diameter of the index tumour, and the T-stage in preoperative PSMA–ligand PET/CT than the CAPRA or D’Amico scores [43]. The CAPRA score evaluates the risk of recurrence in localised PCa using pretreatment factors such as the pretreatment PSA, Gleason score, clinical stage, percentage of positive biopsy cores, and the patient’s age [44]. The D’Amico system classifies patients into risk groups to inform treatment based on the PSA, Gleason score, and clinical stage to predict outcomes and guide treatment decisions [45]. Along with other smaller retrospective cohorts, these studies suggested that SUVMax has potential utility for patients who may experience BCR earlier. Further prospective studies will need to be conducted to validate the current findings [40]. OS was not identified in these retrospective studies, perhaps explained by the short follow-up period. We await the 54-month post hoc analysis of the proPSMA study, which may provide further insights surrounding the preoperative SUVMax and the consequent BCR rates [2].

## 3. PSMA PET Imaging-Directed Therapy and Measures of Treatment Response

### 3.1. PSMA PET/CT Directed External Beam Radiation Therapy (RT)

Studies assessing the role of SUVMax and PSMA’s expression in the impact on definitive RT for localised PCa is limited compared with RP. Most research has focused on PSMA PET/CT and its role in guiding salvage radiation therapy (sRT) [36]. While inferences can be made that poor oncological outcomes may be present in patients with a high SUVMax treated with RT, not all studies have supported this hypothesis [46,47]. Biology-guided radiotherapy (BgRT) enhances the capacity for intensified radiotherapy dosing guided by PET imaging; nonetheless, challenges persist in achieving precise targeting. Higher RT doses may be delivered to sites with increased SUVMax or other aggressive features revealed by PSMA PET/CT, while reducing the dose for other, less aggressive disease locations [48].

Current dose escalation protocols are predominantly based on FDG PET/CT or hypoxia-based tracers [49]. However, a post hoc analysis of the prospective proPSMA study assessed the feasibility of PSMA-guided definitive radiotherapy in a cohort of 84 patients using SUVMax thresholds and strong signal-to-background ratios [48]. Caution against BgRT remains, considering the increased rates of high-grade toxicities [48].

In patients with nodal or local recurrence after RP, PSMA PET/CT SUVMax prior to sRT may be prognostic for future recurrence but is only significant in the setting of local recurrence [40,50,51]. There is potential for SUVMax to further guide intensification or de-escalation of the treatment in patients undergoing sRT therapy in the future; however, there remains a paucity of evidence to support this practice [52,53,54].

### 3.2. PSMA PET/CT and Hormonal Therapy—A Measure of Treatment Response

ARPIs as hormonal treatments appear to upregulate PSMA’s expression or “PSMA flares”, and may have implications in PSMA-based theranostics. In an international retrospective, multicentre analysis of 54 patients with mCRPC, Unterrainer et al. established that PSMA PET/CT SUV parameters, including SUVMax, are not drastically changed within the first 30 days of commencing ARPI treatment, with the inference that PSMA’s expression is not impacted by ARPI at an early time point [34]. The study also indicated that whole-body PSMA PET/CT total tumour volume (TV) increased in the first 30 days. However, in vivo animal studies have demonstrated that perhaps the uptake of PSMA is not correlated with tumour size as measured with callipers [55].

Contrasting results have been demonstrated by Emmett et al., indicating rapid changes to PSMA’s expression at 9, 18, and 28 days, as measured by SUVMax on PSMA PET/CT after commencing ARPIs [56]. Androgen blockade was defined by luteinising hormone-releasing hormone +/− bicalutamide in patients with HSPC, and enzalutamide or abiraterone in CRPC. The study reported a 30% decrease in SUVMax within 9 days of starting treatment and a correlated PSA treatment response in all men. PSMA’s total TV was also reduced. Interestingly, after 9 days, heterogeneity in SUVMax appeared, with some continuing to increase in SUVMax and others experiencing plateaus. These findings further corroborated the complex differences in PCa and may have implications for the timing and sequencing of PSMA-targeted therapies [56].

### 3.3. PSMA PET/CT SUVMax and Taxane Therapy

The role of taxane therapies predominantly lies in mCRPC; however, upfront utilisation of docetaxel in the mHSPC treatment landscape has become widespread. Multiple studies have identified SUVMax and PSMA’s expression as independent poor prognostic factors within mCRPC; however, they may act as a useful therapeutic target, given their ubiquity in the metastasis of PCa [57]. Such findings are concordant with our understanding that SUVMax and PSMA’s expression are generally related to more aggressive disease. Within the context of taxane-based chemotherapy in patients with mCRPC, high SUVMax and expression of PSMA prior to therapy acted as an independent poor prognostic indicator for lower OS [57]. These retrospective findings reported by Vlachostergios et al. further found that a lower OS was independent of the treatment received, including taxanes, ARPI or another type of androgen blockade, and radioligand treatment with radium-223 [57]. As supported by other research, SUVMax was found to be correlated with serum PSA levels [58].

Post-therapy expression of PSMA may also play an important role in assessing the treatment response to taxane-based chemotherapy. Within a retrospective analysis of 8 mHSPC and 29 mCRPC patients treated with docetaxel or cabazitaxel, Shagera et al. highlighted that 18 out of 37 patients had TV responses and the remainder were unchanged [59]. PSMA TV selected by SUVMax as assessment of the overall tumour burden may be reflective of taxane-based chemotherapy’s success. In addition, increased expression of PSMA and TV may also play a role in predicting poor outcomes [60].

## 4. PSMA Radioligand Therapy (RLT)

**Figure 3:**
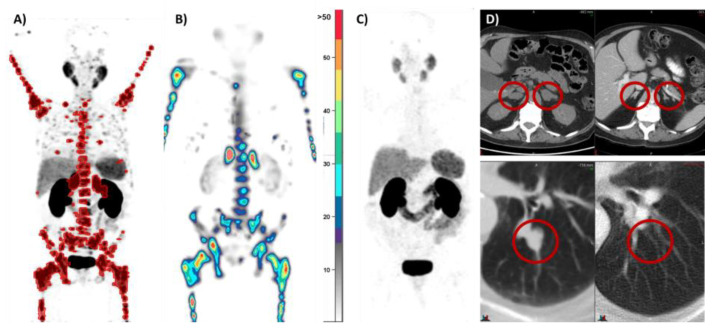
PMCC patient with mCRPC. (**A**) [^68^Ga] Ga-PSMA-11 PSMA PET/CT in a patient after six lines of prior therapy. (**B**) After 8 GBq of [^177^Lu]Lu-PSMA-617: quantitative SPECT/CT demonstrating the delivery of 68 Gy to adrenal metastases, 33 Gy to bone metastases, and <4 Gy to off-target organs (parotid/kidneys). (**C**) Three months after two cycles of [^177^Lu]Lu-PSMA-617: repeat PSMA PET/CT demonstrated a complete response. (**D**) CT also demonstrated a marked response with a reduction in the size of the right adrenal metastasis and resolution of the left adrenal and pulmonary metastases.

Unlike other treatments, [^177^Lu]Lu-PSMA-617 RLT acts as a form of targeted radiotherapy with selective uptake in PSMA-expressing cells (Figure 3) [61]. In turn, the advent of PSMA RLT as a novel form of precision oncological treatment is being validated and adopted into clinical practice [62,63,64].

At the core of a theranostic approach, being able to “see what you treat” allows for precision medicine. [^177^Lu]Lu-PSMA-617 has shown promise in mCRPC patients pretreated with taxane-based chemotherapy and second-generation anti-androgens [29]. Patients who received [^177^Lu]Lu-PSMA-617 achieved a decline in PSA of 50% or more, and had improvements in the severity of pain and interference scores. [^177^Lu]Lu-PSMA-617 was also associated with low toxic effects [29]. Two further large prospective randomised control studies—Phase III VISION and Phase II TheraP—have contributed to the clinical usage of [^177^Lu]Lu-PSMA-617. However, these two studies had varying inclusion criteria based on PSMA PET/CT. VISION utilised a SUVMax threshold of one metastasis or more being greater than the liver’s SUV, whereas TheraP required patients to have a SUVMax of above 20 at one site of the disease and 10 at all other sites. Additionally, patients with discordant disease based on FDG PET/CT and PSMA PET/CT or PSMA-negative disease were excluded in TheraP.

Prior to the VISION trial comparing [^177^Lu]Lu-PSMA-617 with standard care excluding chemotherapy, radiotherapy, radium-223, and other investigational drugs, Hofman et al. reported the clinical efficacy and safety of [^177^Lu]Lu-PSMA-617 [29,62]. In a biomarker analysis of the TheraP cohort by Buteau et al., a PSMA PET/CT SUVMax of greater than 10 was associated with favourable outcomes and increased OS [65]. A metabolic TV greater than 200 mL on FDG PET/CT was found to be a prognostic biomarker regardless of the treatment received [65]. While FDG PET/CT is not routinely performed in all patients globally and was not an exclusion criterion in the VISION trial, the findings of this study allow for potential insights into the sequencing of systemic treatment in mCRPC patients [65].

In the earliest use of RLT in the PCa disease spectrum, the LuTectomy trial, a Phase I/II study, assessed the safety and efficacy of upfront [^177^Lu]Lu-PSMA-617 prior to RP in men with high-risk localised PCa. The authors demonstrated that neoadjuvant LuPSMA delivers high but variable doses of targeted radiation to the sites of tumours that express PSMA. It was well tolerated, and surgery following LuPSMA treatment was safe and viable. Early indicators of oncologic effects, including imaging responses, histological responses, and reductions in PSA showed promise, with a median reduction in PSA of 49%, and 80% of men achieving BCR-FS over a median follow-up of 13.8 months [14].

The current landscape of Lu-PSMA is evolving, with combination therapy taking centre stage. Many assessments of Lu-PSMA utility in conjunction with other agents in varying stages of CaP are demonstrated in Table 1. Other isotopes are being explored as potential therapeutic agents. It should be noted, however, that the large majority of the combination trials are being conducted within an mCRPC setting. Further research should assess the safety and efficacy of LuPSMA in combination with other agents.

Until today, [^177^Lu]Lu-PSMA-617 has been the only PSMA RLT that has received FDA approval for the treatment of PSMA-positive mCRPC following the failure of ARPIs and taxane-based chemotherapy [66]. However, the field is rapidly evolving, and α-ray-emitting radionuclides are under investigation. The use of [^225^Ac]Ac-PSMA-617 was first reported by Kratochwil et al. in 2016, in the setting of aggressive mCRPC that was progressive after conventional therapy and [^177^Lu]Lu-PSMA-617 [67]. Although some reports showed a good PSA response, clinical experience is limited to retrospective observational studies, with no long-term data on progression-free survival (PFS) and OS [68]. In addition, the supply of [^225^Ac]Ac-PSMA-617 is small and there have been concerns due to hematologic toxicity and xerostomia [69,70]. Phase I/II studies with [^225^Ac]Ac-PSMA-617 (AcTION: NCT04597411) and [^225^Ac]Ac-PSMA-617-I and -T (TATCIST: NCT05219500) are currently in course, and will provide more information on the therapeutic efficacy, safety, and dose-limiting toxicity [71]. Other α-ray RLT, [^161^Tb]Tb-PSMA-I and -T (NCT05521412), and [^227^Th]Th-PSMA-I and -T (NCT03724747) therapies are currently being evaluated in Phase I trials [68].

**Table 1:** Landmark PSMA RLT trials at different cancer stages.

Title	Short Title	Trial Registration Code	Intervention	Disease Stage	Author	Trial Design	Primary Endpoint
Administering [^177^Lu]Lu-PSMA-617 Prior to Radical Prostatectomy in Men with High-Risk Localised Prostate Cancer (LuTectomy): A Single-Centre, Single-Arm, Phase 1/2 Study [14]	Lutectomy	NCT04430192	Neoadjuvant [^177^Lu]Lu-PSMA-617 prior to robotic-assisted radical prostatectomy (RARP)	Localised PCa	Eapen	Single-centre, single-arm Phase I/II study	Dose of radiation absorbed by the tumour
Neoadjuvant ^177^Lu-PSMA-I&T Radionuclide Treatment in Patients with High-risk Prostate Cancer Before Radical Prostatectomy: A Single-Arm Phase 1 Trial [72]	NaLuProst	NCT04297410	Neoadjuvant [^177^Lu]Lu-PSMA-I and -T prior to RARP	Localised PCa	Golan	Open-label, single-arm clinical trial	Safety, as defined by the rate of perioperative complications and the rate of functional toxicity
177-Lutetium–PSMA Before Stereotactic Body Radiotherapy for the Treatment of Oligorecurrent Prostate Cancer (active)	LUNAR	NCT05496959	[^177^Lu]Lu-PSMA-617, stereotactic ablative radiotherapy (SABR)	Oligorecurrent PCa	Kishan	Randomised prospective Phase II clinical trial	PSMA PET/CT radiological PFS
LuPSMA for Oligometastatic Prostate with STereotactic Ablative Radiotherapy: A Randomised Phase II Parallel Cohort Trial (POPSTAR II) (active)	POPSTAR II	NCT05560659	[^177^Lu]Lu-PSMA-617, SABR	Oligometastatic PCa, mHSPC	Siva	Randomised prospective multicentre Phase II trial	Evaluating the PFS of SABR alone and SABR + ^177^Lu-PSMA
In Men With Metastatic Prostate Cancer, What is the Safety and Benefit of Lutetium-177 PSMA Radionuclide Treatment in Addition to Chemotherapy? (UpFrontPSMA) (active)	UpfrontPSMA	NCT04343885	[^177^Lu]Lu-PSMA-617, docetaxel	Metastatic hormone-naive PCa	Azad	Randomised, two-arm, multicentre, Phase II clinical trial	Undetectable rate of PSA at 12 months after commencement of the protocol’s therapy
[^177^Lu]-PSMA-617 Radionuclide Treatment in Patients with Metastatic Castration-Resistant Prostate Cancer (LuPSMA trial): A Single-Centre, Single-Arm, Phase 2 study [29]	LuPSMA	ANZCTR 12615000912583	[^177^Lu]Lu-PSMA-617	mCRPC	Hofman	Single-arm, single-centre Phase II trial	PSA-50 response rate
Lutetium-177–PSMA-617 for Metastatic Castration-Resistant Prostate Cancer [62]	VISION	NCT03511664	[^177^Lu]Lu-PSMA-617	mCRPC	Sartor	International open-label Phase III trial	Imaging-based PFS and OS
[^177^Lu]Lu-PSMA-617 Versus Cabazitaxel in Patients with Metastatic Castration-Resistant Prostate Cancer (TheraP): A Randomised, Open-Label, Phase 2 trial [63]	TheraP	NCT03392428	[^177^Lu]Lu-PSMA-617	mCRPC	Hofman	Randomised, multicentre, unblinded Phase II trial	PSA-50 response rate
Prospective Phase 2 Trial of PSMA-Targeted Molecular Radiotherapy with ^177^Lu-PSMA-617 for Metastatic Castration-Resistant Prostate Cancer (RESIST-PC): Efficacy Results of the UCLA Cohort [73]	RESIST-PC	NCT03042312	[^177^Lu]Lu-PSMA-617	mCRPC	Calais	Randomised prospective multicentre Phase II trial	PSA-50 response rate
^177^Lu-PSMA-617 vs. Androgen Receptor-Directed Therapy in the Treatment of Progressive Metastatic Castration-Resistant Prostate Cancer (PSMAfore) (active)	PSMAfore	NCT04689828	[^177^Lu]Lu-PSMA-617	mCRPC or mHSPC in the taxane-naive setting	Sartor	Randomised open-label, multicenter Phase III clinical trial	Radiographic PFS
Enzalutamide With Lu PSMA-617 Versus Enzalutamide Alone in Men with Metastatic Castration-Resistant Prostate Cancer (ENZA-p) (active)	Enza-P	NCT04419402	[^177^Lu]Lu-PSMA-617, enzalutamide	mCRPC	Emmett	Randomised prospective two-arm, multicentre Phase II clinical trial	PSA PFS
Evaluation of Radioligand Treatment in men with Metastatic Castration-Resistant Prostate Cancer With [^161^Tb]Tb-PSMA-I&T (VIOLET) (active)	VIOLET	NCT05521412	[^161^Tb]Tb-PSMA-I and -T	mCRPC	Buteau	Prospective single-centre, single-arm, open-label Phase I/II trial	Dose-limiting toxicities, maximum tolerated dose, recommended Phase II dose, PSA-50 response rate
Cabazitaxel in Combination with ^177^Lu-PSMA-617 in Metastatic Castration-Resistant Prostate Cancer (LuCAB) (active)	LuCab	NCT05340374	[^177^Lu]Lu-PSMA-617, cabazitaxel	mCRPC	Kostos	Prospective single-centre, single-arm, open-label Phase I/II trial	Dose-limiting toxicities, maximum tolerated dose, recommended phase II dose
Combination of Radium-223 and Lutetium-177 PSMA-I&T in Men with Metastatic Castration-Resistant Prostate Cancer (AlphaBet) (active)	Alphabet	NCT05383079	[^177^Lu]Lu-PSMA-I and -T, radium-223	mCRPC	Kostos	Prospective single-centre, single-arm, open-label Phase I/II trial	Dose-limiting toxicities, maximum tolerated dose, recommended Phase II dose, 50% PSA response rate
Single-Dose ^177^Lu-PSMA-617 Followed by Maintenance Pembrolizumab in Patients with Metastatic Castration-Resistant Prostate Cancer: An Open-Label, Dose-Expansion Phase 1 Trial (active)	N/A	NCT03805594	[^177^Lu]Lu-PSMA-617, maintenance pembrolizumab	mCRPC	Aggarwal	Open-label, prospective, dose-expansion Phase I study	Part A: Phase II dose schedule of the treatment combination. Part B: objective response rate per investigator assessment by RECIST
PSMA-Lutetium Radionuclide Therapy and Immunotherapy in Prostate Cancer (active)	PRINCE	NCT03658447	[^177^Lu]Lu-PSMA-617, pembrolizumab	mCRPC	Sandhu	Prospective single-centre Phase Ib/II study	PSA-50 response rate
Phase II Study of Radionuclide ^177^Lu-PSMA Therapy versus ^177^Lu-PSMA in Combination with Ipilimumab and Nivolumab for Men with Metastatic Castration-Resistant Prostate Cancer (mCRPC) (active)	Evolution	NCT05150236	[^177^Lu]Lu-PSMA-617, nivolumab, ipilimumab	mCRPC	Sandhu	Randomised prospective, multicentre Phase II study	PSA PFS at 1 year
177 Lu-PSMA-617 Radioligand Therapy of Metastatic Castration-Resistant Prostate Cancer: Initial 254-Patient Results from a Prospective Registry (REALITY study) [74]	REALITY	NCT04833517	[^177^Lu]Lu-PSMA-617	mCRPC	Khreish	Registry-based study	PSA PFS, OS, caregiver-reported and patient-reported safety response to RLT
Prostate Cancer Theranostics and Imaging Centre of Excellence Compassionate Access Registry (active)	ProsTIC registry	NCT04769817	[^177^Lu]Lu-PSMA-617	mCRPC	Hofman	Registry-based study	N/A
^177^Lu-PSMA-617 Therapy and Olaparib in Patients with Metastatic Castration Resistant Prostate Cancer (LuPARP) (active)	LuPARP	NCT03874884	[^177^Lu]Lu-PSMA-617, olaparib	mCRPC	Sandhu	Open-label, multicentre, dose-escalation and dose-expansion Phase I study	Dose-limiting toxicities, maximum tolerated dose, recommended Phase II dose

RARP, robotic-assisted radical prostatectomy; PCa, prostate cancer; PFS, progression-free survival; SABR, stereotactic ablative radiotherapy; mHSPC, metastatic hormone-sensitive PCa; mCRPC, metastatic castration-resistant PCa; OS, overall survival.

## 5. Conclusions

The pathological manifestation of prostate-specific membrane antigen (PSMA) in prostate cancer cells has played a pivotal role in revolutionising the diagnostic and therapeutic landscape of prostate cancer. PSMA-targeted positron emission tomography (PET) tracers have significantly enhanced the precision of diagnosing metastasis at an earlier timepoint. Radiomic evaluation of radiotracer uptake in PET has allowed for significant advancements in the interpretation of intraprostatic and metastatic disease.

Beyond its diagnostic utility, PSMA holds promise as a biomarker, with implications for prognosis and predicting responses to treatment. The prospect of tailoring and sequencing treatment regimens based on the levels of PSMA’s expression presents an avenue for optimising patient outcomes. However, due to the heterogeneity of PSMA’s expression, particularly in advanced disease settings, further assessments within real-world contexts are necessary.

Looking ahead, the focus on PSMA may extend to elucidating the heterogeneity within prostate cancer. A comprehensive understanding of PSMA’s expression patterns across disease stages is imperative for the development of targeted interventions. The integration of PSMA-based analysis with emerging technologies, such as artificial intelligence and genomic profiling, represents a promising approach for achieving a more personalised and nuanced strategy in prostate cancer care. The utility of PSMA’s expression is focused on improving patient outcomes by providing more precise diagnostic and treatment pathways.

## Abbreviations

ARPIs	Androgen receptor pathway inhibitors
AS	Active surveillance
BCR	Biochemical recurrence
BCR-FS	Biochemical recurrence free survival
BgRT	Biology guided radiotherapy
CI	Conventional imaging
csPCa	Clinically significant prostate cancer
EANM	European Association of Nuclear Medicine
EAU	European Association of Urology
ePLND	Extended pelvic lymph node dissection
GBq	Gigabecquerel
GGG	Gleason grade group
Gy	Gray
IHC	Immunohistochemical
ISUP	International Society of Urological Pathologists
LN	Lymph node
MRI	Magnetic resonance imaging
mCRPC	Metastatic castration-resistant prostate cancer
mHSPC	Metastatic hormone-sensitive prostate cancer
OS	Overall survival
PCa	Prostate cancer
PFS	Progression-free survival
PMCC	Peter MacCallum Cancer Centre
PIRAD	Prostate Imaging–Reporting and Data System
PSA	Prostate-specific antigen
PSMA	Prostate-specific membrane antigen
RLT	Radioligand therapy
RARP	Robotic-assisted radical prostatectomy
RP	Radical prostatectomy
RT	Radiation therapy
sRT	Salvage radiation
SUV	Standard uptake value
SUVMax	Maximum standard uptake value
TV	Tumour volume
